# From far-infrared detectors to THz QCLs

**DOI:** 10.1515/nanoph-2025-0221

**Published:** 2025-07-28

**Authors:** Qing Hu

**Affiliations:** Department of Electrical Engineering and Computer Science and Research Laboratory of Electronics, 2167 Massachusetts Institute of Technology, Cambridge, MA 02139, USA

**Keywords:** THz, terahertz, QCL, quantum cascade lasers, far-infrared

## Abstract

On this special occasion as we celebrate the 30th anniversary of quantum cascade lasers (QCLs), the author would like to reflect his own journey from the previous research on far-infrared superconducting devices to the development of THz QCLs. The initial phase of this journey spanned over 12 years (1990–2002) till we finally reached the lasing threshold, and it involved three generations of graduate students (Jurgen Smet, Bin Xu, and Ben Williams). The journey was not totally smooth, but full of joy from learning new things that are quite different from the author’s previous background to the eventual witness of laser operation of the very first THz QCL developed in the author’s group. This article is mostly a memoir of the author’s own process in the development of THz QCLs with many unpublished episodes, and it is by no means intended as a review article of the overall field. As such, work from other groups working in the field will not be cited thoroughly.

## 1 Introduction

The author did his Ph.D. research on nonlinear dynamics of Josephson junctions driven by a far-infrared gas laser. As the laser frequency is close to the Josephson plasma frequency, Josephson junctions can be driven into chaotic regime through period doubling bifurcation [1]. The phenomenon of nonlinear dynamic is quite rich and fascinating, as a small change of the driving laser intensity would push the device into the chaos regime from a normally well behaved AC Josephson effect exhibited as Shapiro steps. After Ph.D., the author went to U. C. Berkeley to pursue a postdoc in Prof. Paul Richards’ group, and the project was to develop SIS (superconductor–insulator–superconductor) mixers at submillimeter-wave (far-infrared) frequencies [2]. When the author joined the faculty of Electrical Engineering and Computer Science at MIT in January 1990, he naturally continued the work at Berkeley and pushed the SIS operating frequencies to ever higher towards 1 THz. This was highly challenging then as the *RC* roll-off becomes severe as the frequency is increased above 100 GHz. More challenging for actual applications, however, the author realized that compact solid-state local oscillators (LOs) were not available above 0.5 THz. The lack of LOs in the THz frequency range intrigued the author, who as a recently minted faculty member was actively looking for new research projects/directions.

It was well known that a “THz gap” exists for semiconductor radiation sources and amplifiers. Semiconductor electronic devices, such as transistors, work well at low frequencies; while conventional semiconductor photonic devices, such as laser diodes, work well at high frequencies up to visible. Only much later the author understood the physical origin of this so-called THz gap. Among the four Maxwell’s equations, only the modified Ampere’s law contains sources ( $\nabla \times \overset{⇀}{H}=\overset{⇀}{J}+\frac{\partial \overset{⇀}{p}}{\partial t}+\varepsilon_{o}\frac{\partial \overset{⇀}{E}}{\partial t}$ ), and therefore it is responsible for the generation of electromagnetic waves. The conduction current $\overset{⇀}{J}$ is produced by freely moving electrical charges (electrons), and as such, it is subject to the limitations of transit time and *RC* roll-off. Above the roll-off frequencies of these two limitations, the amplitude decreases with frequency as *J* ∝ 1/*f* ^2^ and correspondingly the power drops with frequency as 1/*f* ^4^. All semiconductor electronic devices function by manipulating $\overset{⇀}{J}$ and therefore their power levels drops as 1/*f* ^4^ with the frequency approaching and above 1 THz. Semiconductor photonic devices, on the other hand, function based on the term $\frac{\partial \overset{⇀}{p}}{\partial t}$ . That is, they generate electromagnetic radiation by oscillating dipoles, or more precisely, by oscillation of bounded electrons. As localized charge neutral dipoles, clearly, they do not suffer the aforementioned two limitations, since they do not travel in real space and they do not charge and discharge capacitors. As a result, semiconductor photonic devices can function at very high frequencies. However, the oscillating dipoles are generated by quantum mechanical transition between two energy levels. In semiconductors, these two levels correspond to the edges of conduction band and valence band. The narrowest bandgap of natural semiconductor in lead salt is ∼40 meV, or 10 THz. Based on this fundamental argument, the THz gap in the frequency range of 1–10 THz seemed to be insurmountable so is there any way out?

The above discussion is correct but oversimplistic. Additional and deeper insight into the physical origin of the *RC* and transit-time roll-off was only developed much later (∼2012) by the author. It is well known in solid-state physics that the dielectric constant *ε*(*ω*) and electric conductivity *σ*(*ω*) are totally equivalent in describing time-harmonic polarization except at *ω* = 0. Consequently, there is no rigid distinction between $\overset{⇀}{J}$ and $\frac{\partial \overset{⇀}{p}}{\partial t}$ . Whether a device is subject to the *RC* roll-off is determined by whether the moving charge travels through the medium and reaches *both* terminals of the device within one cycle. If it does, it will generate current in the external circuit that connects the two terminals, and therefore the device is subject to the *RC* roll-off due to the charging and discharging of the capacitor. If not, no external current is generated and thus there is no *RC* roll-off. An illustrative example is the photon-assisted tunneling (PAT) in superconductor–insulator–superconductor(SIS) tunnel junctions [2]. Even though PAT is a purely quantum mechanical process, the charges (quasiparticles) do oscillate through the tunneling barrier and therefore the process is subject to the *RC* roll-off. As for the transit-time roll-off, it is more informative to understand it in the frequency domain. The complex electric conductivity is $\sigma \left(\omega \right)=\sigma^{\prime }+i\sigma^{\prime \prime }$ and in Drude model, $\sigma^{\prime }=\frac{ne^{2}\tau }{m\left[1+{\left(\omega \tau \right)}^{2}\right]}$ . The area under $\sigma^{\prime }\left(\omega \right)$ is $\int_{0}^{\infty }\sigma^{\prime }\left(\omega \right)d\omega =\frac{\pi }{2}\frac{ne^{2}}{m}$ , which is independent of *τ*. It can be shown from Kramers–Kronig relation (Kittel p314) that this relation is model independent and is the result of oscillator strength sum rule. This sum rule can be used to understand the infinite dc conductivity in superconductors. In the superconducting state, $\sigma^{\prime }\left(\omega \right)=0$ in the frequency range below the energy gap Δ/*ℏ* except at *ω* = 0. However, the “missing area” of the oscillator strength is not actually missing. Instead, it is condensed into a single frequency *ω* = 0, yielding $\sigma^{\prime }\left(\omega \right)=A\delta \left(\omega \right)$ where *A* is the missing area, hence the perfect conductivity at dc [3]. In the current context, for freely moving charges (no springs attached), most of the oscillator strengths are concentrated in low frequencies, hence the roll-off at high frequencies. In contrast, for bounded electrons, much of the oscillator strengths are shifted to higher frequencies near the resonance of electronic motion, eliminating the transit-time issue. This detailed analysis favors quantum electronic approaches (for example, lasers), which are free of the *RC* and transit-time limitations, to develop sources in the THz-gap frequency range. The key challenge is to develop quantum mechanical systems with energy separations corresponding to THz frequencies.

## 2 Discussions

It had been known for some time that semiconductor quantum devices based on size-dependent quantum effects are human-made quantum mechanical systems, in which the energy levels can be designed and engineered to be any value [4]. Transitions between those engineered energy levels (intersubband transitions) had already been utilized in the successful development of QWIPs (Quantum well intersubband photodetectors) [5]. As a very fortunate timing for the author, emitters based on the intersubband transitions were just demonstrated. In the 1990 APS (American Physical Society) March meeting, Dr. Manfred Helm from Bellcore gave an invited talk on this ground-breaking result. The author attended that invited talk, and needless to say, was blown away by the concept and result. The author was especially intrigued by Dr. Helm’s argument that lasers could be developed based on intersubband transitions, and THz range is preferable since the intersubband transition energy is below that of the longitudinal optical (LO) phonons. Therefore, the electrons residing in the upper state would have a long lifetime, facilitating the population inversion between two subbands [6].

**Figure 1:**
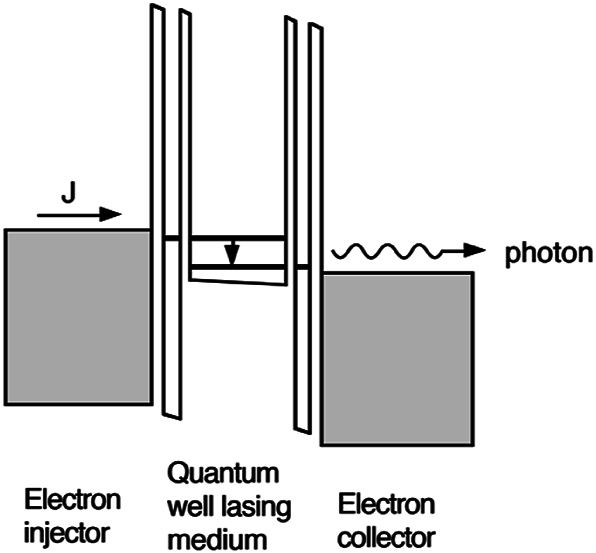
Schematic of proposed interband THz laser. Population inversion is facilitated through resonant tunneling into the upper subband and selective extraction of the lower subband [7].

The author quickly selected the development of lasers based on intersubband transitions as a new research direction, and the first step was to learn more about lasers and semiconductors. Both were not familiar subjects to the author then, even though he used far-infrared gas laser in his Ph.D. research. Developing lasers, especially a new type that did not exist then was a totally a new discipline for the author. In the summer of 1990, the author read through the book “Quantum Electronics” by Amnon Yariv multiple times, while performing many typical tasks as a new faculty member, such as writing proposals, lab renovation, and recruiting and training new students. With a fruitful collaboration with the author’s close friend/colleague, the late Prof. Shechao Feng, we co-authored a paper on the design analysis of intersubband lasers [7]. A schematic of the proposed structure is shown in Figure 1. Prior to this paper, several papers were already published on similar subject [8], [9], [10]. Although all the proposed schemes in these papers do not closely resemble the eventual working QCLs, they did provide early conceptual development that laid the ground for later successful development. The new contribution in [7] is to use plasmas in heavily doped contact layers to confine the mode within the gain medium. Only much later when we realized that the heavy loss in the plasma layers will make the lasing threshold prohibitively high.

The first-generation student working on this project, Jurgen Smet, energetically pursued both experimental and theoretical investigations. At that time, we simply did not know the relative energy alignment of subband levels. Performing far-infrared emission spectroscopy was only achieved much later. Smet came up with a clever experiment to measure the intersubband level spacing by performing magneto-tunneling spectroscopy on a device grown using MBE in Prof. Clif Fonstad’s group at MIT [11]. The de Hass-Shubinikov oscillation gives an accurate measure of the intersubband spacing as a function of bias. This measurement validated the material parameters and numerical codes that were used in the design. Later, Smet expanded the numerical code to include gain and various scattering processes [12]. The key finding of this work is that electron–electron (e–e) scattering dominates the nonradiative intersubband transitions at THz frequencies. Electron-LO phonon scattering is energetically forbidden for THz intersubband structures at low temperatures, and the electron-acoustic phonon scattering is quite slow with a lifetime ∼1 ns. Hence, without e–e scattering electrons should have a long lifetime (∼1 ns) staying in the upper subband, and consequently the lasing threshold should be easily reached at low injection current densities. However, experimental reality told a completely different story, and ignoring e–e scattering in all the previous work was responsible for this discrepancy.

In 1994, the Bell lab group led by Federico Capasso made a breakthrough in the first development of lasers based on intersubband transitions (Quantum cascade lasers, QCLs) [13]. The lasing wavelength of 4.2 µm corresponds to ∼70 THz, which is far above the THz band that we all thought would be the preferable frequency range for intersubband lasers. In a later private communication with the leading author (Faist), we learned that one of their key revelations is that if they move the lasing frequency far above the LO-phonon energy *ℏω*_*LO*_ ≈ 9 THz, the LO-phonon scattering time actually increases from that at the resonance. Therefore, if the lower lasing level is placed at *ℏω*_*LO*_ above the ground state, then it will have a (much) shorter lifetime and population inversion can then be established. Once LO-phonon scattering becomes energetically allowed, the aforementioned e–e scattering becomes negligible. The community later learned that population inversion is easier to achieve and maintain at infrared than at THz, a realization that is totally opposite from the initial projection.

In 1994, a new student (Bin Xu) joined the author’s group. Inspired by the Bell Lab’s breakthrough, Xu took on the project with enthusiasm and gusto. He modified a numerical code package, originally developed at Purdue University, to design intersubband emitters. At the time we also got MBE wafers from Prof. Mike Melloch’s group at Purdue University. Built on top of Smet’s magneto-tunneling spectroscopy, we tried to perform far-infrared emission spectroscopy to further validate the result and to provide the first step towards developing lasers. Before then, all the far-infrared emission measurements were done using cryogenic detectors coupled through light pipe to the emitter [6]. A filter whose absorption frequency can be tuned with a magnetic field (cyclotron frequency) is placed before the detector, hence spectroscopy with low resolution can be performed. The Bell lab group used a free space Fourier transform infrared spectrometer (FTIR) to measure the lasing spectra. FTIR has a high resolution, limited only by the mirror traveling distance. It would be a big leap in the field to measure THz radiation (nonlasing) in free space using a FTIR. Prior to the development of QCLs, the Bell lab group did measure the intersubband spontaneous emission using FTIR [14]. The power level at infrared is much higher than at THz because (1) the transition rate scales as *ω* ^2^, and (2) the photon energy *ℏω* is much greater. The first challenge is to align the invisible and weak THz radiation with the FTIR, for which we benefitted from suggestion given by Dr. Carlo Sirtori then at Bell Lab [15]. Once we have made a good alignment, we were able to perform spectroscopy on weak THz spontaneous emission at power levels of ∼100 pW [16], although we later realized that the measured signals may be heavily contaminated by blackbody radiation from the heated devices, even though they were submerged in liquid helium.

**Figure 2:**
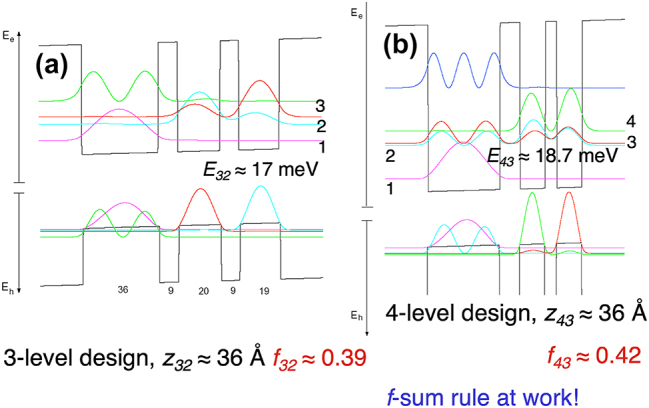
Schematics of interband-pumped THz lasers. (a) A three-level system and (b) a four-level system.

In parallel with Bin Xu’s work, another student Ilya Lyubomirsky pursued THz emitter scheme based on intersubband optical pumping [17], [18]. The experiment was much more difficult than that for the electrically pumped THz emitters, since two simultaneous optical alignments are required for both the input and output. Nevertheless, this investigation led to a later scheme using interband optical pumping, as described in Figure 2.

In 1998, the third-generation student Ben Williams took over the project. He first discovered a highly counter-intuitive result. As he increased the pulse duration of the device bias, the measured average power actually decreased. Only after much analysis we realized that the heat removal by submerging the device in liquid helium was totally inadequate. The heated device created helium gas bubbles around it, which significantly reduces the effectiveness of heat removal, hence the counter-intuitive result. Ben then redesigned the device mount, more in line with a laser mount as opposed to the cooling scheme of Josephson junctions that the author used in his Ph.D. research. With the adequate heat removal, we finally saw unambiguous intersubband emission at THz, with a linewidth as narrow as 2 meV [19].

In parallel with the development of electrically pumped THz QCLs, we also pursued theoretical investigation of optically interband pumped THz intersubband laser, whose schematics are shown in Figure 2 [20]. Clearly, the three-level scheme shown in (a) has a much greater spatial overlap between the lasing levels 3 and 2 than the four-level system in (b) between the lasing levels 4 and 3. However, the computed oscillator strength of the latter scheme is greater (0.42 vs. 0.39). We initially thought this was an error in the computer simulation. After double and triple checking, the error was nowhere to be found so the result is real. Only after much further theoretical investigation, we realized that this apparent counter-intuitive result stems from oscillator strength sum rule. The rule states that the sum of the oscillator strengths of dipole transition from an eigenstate of a Hermitian Hamiltonian to all the other eigenstates is identical to unity. As a result, since there are two levels below the lower lasing level 3 in (b), the oscillator strength of its transition to upper level (with oppositive sign from the downward transitions to 2 and 1) is boosted. This conceptual revelation led to the final realization of resonant-phonon scheme of THz QCLs.

From our very early design of THz QCLs [16], we followed the design principle of the Bell Lab group on the IR QCLs by using the fast LO-phonon electron scattering to depopulate the lower lasing level. The easiest scheme is to use the three-level system in Figure 2(a). However, this scheme has little selectivity in the depopulation process. If the barrier between levels 2 and 1 is made thin, then the lower-state depopulation time *τ*_2_ can be short but so will be the upper-state lifetime *τ*_3_, which will make establishing population inversion between levels 3 and 2 difficult. In contrast, for the first IR QCLs [13], because of the large energy difference between *E*_3_ − *E*_2_ and *E*_2_ − *E*_1_, the upper-state lifetime *τ*_3_ is much longer than *τ*_2_, which is favorable in establishing population inversion between 3 and 2. On the other hand, the resonant-phonon scheme in Figure 2(b) is much more advantageous in this aspect. Because the levels 3 and 2 are in resonance, both are spatially extended into the wide phonon well where the ground state level 1 resides. The fast depopulation through LO-phonon scattering can take place there. On the other hand, the upper state level 4 resides mostly away from the phonon well so it will have a much longer scattering time to level 1. In combination of the aforementioned greater oscillator strength, this resonant-phonon scheme naturally became our choice to pursue THz QCLs.

The subband wavefunctions in Figure 2 were calculated in a totally coherent picture, in which dephasing due to various scattering mechanisms is ignored. As a result, the wavefunctions of levels 3 and 2 are extended at their resonance (at anticrossing). In reality, it is known that dephasing is significant in solid-state devices, and if it is so strong that the coupling between levels 3 and 2 behaves like an overdamped oscillator [21], then the wavefunction of level 3 will be localized in the double-well region where level 4 resides, while level 2 will be localized in the wide phonon well. In this scenario, the aforementioned high selectivity in depopulation will be severely diminished. In order to investigate the effect of dephasing and also the relative level alignment, we performed magneto-tunneling spectroscopy [22] like what we did in Ref [11]. The result of this investigation was very encouraging that an anticrossing gap as narrow as 1.7 meV can be resolved. This implies that so long as we design the anticrossing gap between levels 3 and 2 to be much greater than 2 meV (∼5 meV in our first successful THz QCL), the coherent picture in Figure 2 holds and we can selectively depopulate the lower lasing level.

In late 2001, as we were pursuing our development of resonant-phonon THz QCLs, words came from Europe that a group led by Prof. Alessandro Tredicucci had made the breakthrough in the development of THz QCLs [23]. This news came as a surprise and shock, since Prof. Tredicucci only recently started working in THz frequency range. Based on this news, we tried to duplicate their THz QCL structure from an earlier publication. We correctly speculated that the structure is based on interminiband transition on which the group performed extensive Monte-Carlo simulation [24]. We copied that design and had our collaborator Dr. John Reno grow it and processed into a metal-metal waveguide structure, but the device failed to lase. We tried two more times and even used the semi insulating surface plasmon(SISP) waveguide as we learned later from the published result [25], but failed in both times. We were really puzzled by this repeated failure until later when we tried to duplicate the THz QCL structure developed at Jerome Faist’s group [26]. Our grown structure not only failed to lase, but when we performed spontaneous emission measurement, the center frequency was ∼1 THz below the lasing frequency demonstrated in [26]. This result gave us the hint that the very thin barriers in the superlattice structures may have not been properly grown, because the shutter may have not been moving fast enough. The author consulted with Dr. John Reno and he increased the shutter speed in his MBE machine. In the following batch, our fourth try to duplicate the result in [25] was finally successful [21], after our own design based on the resonant-phonon scheme also achieved lasing, as discussed in the following.

In the early part of the year 2002, we tried two designs (FL125 and FL175B) of the resonant-phonon scheme and got encouraging results with a spontaneous emission linewidth as narrow as 4 meV measured from edge emitting. The earlier 2-meV linewidth was measured using surface emitting facilitated by a 2nd-order diffraction grating [19], which may have artificially narrowed the linewidth. However, lasing was not achieved using either metal–metal or SISP waveguides. Drawing the aforementioned lesson learned earlier that thin barriers were not grown as designed and the interface roughness scattering (not included in Figure 2) may broaden gain and increase the lasing threshold, we lowered the barrier height from 30 % Al concentration as we have been using since [16] to 15 % Al concentration used in [25], [26]. The logic behind this is that for the same anticrossing gap, halving the barrier height will double the barrier width, lessening the demanding requirement of growing thin barriers. Furthermore, for the unavoidable interface roughness on the order of monolayer, doubling the barrier thickness will relatively reduce the effect of interface roughness scattering.

**Figure 3:**
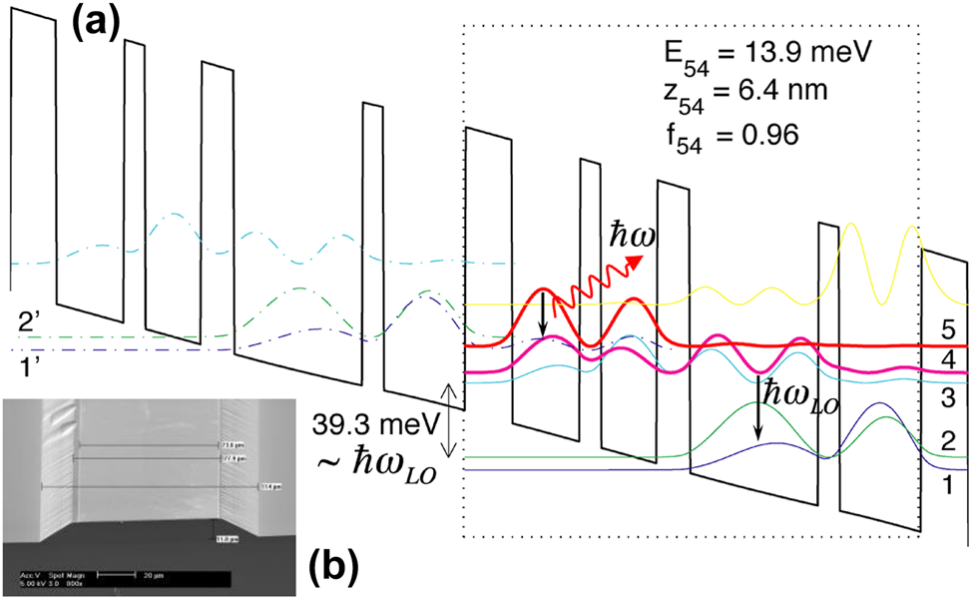
Structures of the THz QCL. (a) Schematic of FL 175C design. The lasing transition is vertical between levels 5 and 4, and the lower level 4 is depopulated through resonant-phonon scheme to the doublet levels 1 and 2. (b) SEM of the SISP waveguide fabricated using wet etching [21].

**Figure 4:**
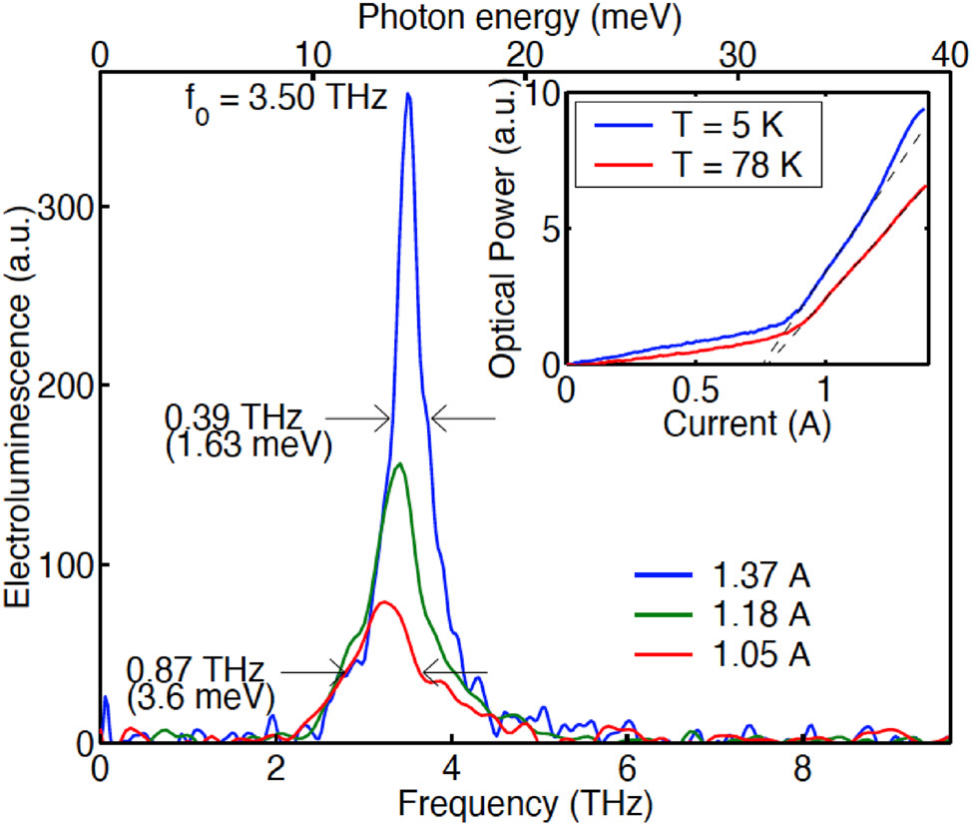
Emission spectra at different bias. The insert shows light-current (L–I) curves measured at 5 K and 80 K.

In the following design of FL175C shown in Figure 3(a), we lowered the barrier height to 15 % Al concentration, and as a result, the thinnest barrier (which is called radiative barrier, the one in the radiative region where levels 5 and 4 overlap in Figure 3(a)) was increased from 5 monolayers (ML) in FL125 to 9 ML. This redesign immediately yielded extremely encouraging result, as shown in Figure 4. In the insert in Figure 4, the light-current (L–I) curve measured at 5 K shows a super-linear behavior. That is, the emission power increases faster than linear with the bias current. Such a super-linear behavior was observed before, but it was attributed to heating with associated broad emission spectra [27]. In contrast, the emission linewidth in Figure 4 shows a clear narrowing with an increased bias. These two features together: super-linear L–I and narrowing linewidth with an increased bias, clearly indicate that the device was approaching the lasing threshold.

The device measured in Figure 4 was 1-mm long without any facet coating. The mirror loss of the cavity is given by: $\alpha_{m}=\frac{1}{2L}\ln\left(\frac{1}{R_{1}R_{2}}\right)$ . We estimated that if we coat the rear facet with high reflection (HR) coating (making *R*_2_ ≈ 1), then the mirror loss will be reduced by ∼6 cm^−1^, which should bring us even closer to the lasing threshold. We did just that over a weekend and started doing measurement on Monday, November 18, 2002. That day/date holds a special place in the author’s memory. The author was fully occupied in teaching in the morning. In the afternoon, the author’s long-time friend/colleague Dr. John Martinis (then at NIST) was to give a seminar at MIT on his work on quantum computing. The author planned to attend this seminar, but decided to drop by the lab on his way to the seminar. Three group members then, Ben Williams, Hans Callebaut, and Sushil Kumar, were glued onto a computer monitor. The author asked Ben Williams if they saw anything interesting, who responded yes, and then added that the power was increased by orders of magnitude. Before that day, the author’s group never had a laser device to measure. The weak power (∼100 pW) of spontaneous emission requires a long integration time of lock-in amplifier when the FTIR was operated in step-scan mode. Even for a low resolution of 4 cm^−1^, it would take an hour to complete a scan. We were still following the same procedure as prior measurements by using step-scan. Remarkably, the interferrogram, instead of decaying quickly as for spontaneous emission, just kept going sinusoidally indefinitely. Even with minimum pause time at each step, the step scan still took hours to complete for the maximum 0.125 cm^−1^ resolution. All four of us just stared at the computer monitor to witness the completion of this step scan, which showed no decay at all in the interferrogram. Once the scanning was completed, Fourier transform showed a narrow-line emission at 3.4 THz with the linewidth limited by the instrument resolution [28]. Remarkably, the emission spectra also showed narrow peaks at 6.8 THz and even higher frequencies. At that time, the Bell Lab group was actively pursuing second-harmonic frequency generation using nonlinear effect in IR QCLs [29]. We thought it would be too big a leap for our first laser to generate second harmonic, so carefully analysed the data. It turned out that because of the weak spontaneous emission, we had been using cryogenic detectors (helium-cooled bolometers or Ga:Ge photodetectors). These cryogenic detectors are very sensitive but have a limited dynamic range. The much higher power levels of the laser (by ∼8 orders of magnitude from ∼100 pW to ∼10 mW) saturated the detector, resulting in an interferrogram clamped at the top of an otherwise pure sinusoid. Hence the second and higher harmonics appeared in the spectra. After identifying the cause, the solution was quite easy. We just punch a small pinhole on an aluminum foil and used it to cover the detector input window, then we obtained clean lasing spectra centered at 3.4 THz. Laer, we just use a room-temperature pyroelectric detector for laser measurements. The pyroelectric detector has a much lower sensitivity than photodetectors, but has a much higher saturation level, so it is operated in linear regime under laser illuminations.

## 3 Postscript

The memorable day of November 18, 2002 marked the beginning of our group’s productive journey in the development of high-performance THz QCLs and their applications in imaging and sensing. In 2003, we integrated the THz gain medium with metal–metal waveguides developed earlier [30]. The metal-metal waveguides can work at arbitrarily long wavelengths, and the tight mode confinement and low waveguide loss significantly reduces the lasing threshold [31]. With the combination of resonant-phonon scheme and metal–metal waveguids, the maximum operating temperature *T*_max_ of THz QCLs was increased to 200 K in 2012 [32]. After that, direct-phonon scheme was explored. In this scheme, both the lower lasing level and the ground state reside in the same wide phonon well and no extraction level (level 3 in Figure 3(a)) was needed. This avoids the complication of double resonance (1′–5 and 4–3 simultaneously at a single bias), which becomes difficult to achieve with heavy doping [33], [34]. The direct-phonon scheme yielded the simpliest QCL structure with only two wells per module [35], [36], [37]. With the direct-phonon scheme and tall barriers (25–30 % Al concentration), *T*_max_ was raised to 210 K in 2019 [38], 250 K in 2020 [39], and 261 K in 2023 [40]. With the QCL gain media, THz radiation amplifier [41] and THz frequency combs [42] were developed. Using a high-power THz QCL and a focal-plane array camera, real-time THz imaging was performed, that is, making movies using T-rays [43]. In collaboration with other groups, dual-comb THz spectroscopy was demonstrated [44], and local oscillators of THz heterodyne receivers were developed for astrophysics studies on a suborbital THz observatory [45].
